# Risk factors and outcomes of critically ill pregnant COVID-19 patients: Experience from the first and second waves of the pandemic

**DOI:** 10.2478/jccm-2025-0008

**Published:** 2025-01-31

**Authors:** Dita Aditianingsih, Noor Hafidz, Aino Nindya Auerkari, Zarah Tin Cahyaningrum, El Nissi Leonard, Chrisella Annabelle

**Affiliations:** Department of Anesthesiology and Intensive Care, Cipto Mangunkusumo Hospital, Faculty of Medicine, Universitas Indonesia, Indonesia; Department of Anesthesiology and Intensive Care, Universitas Indonesia Hospital, Indonesia

**Keywords:** COVID-19, ICU, maternal outcomes, pregnancy

## Abstract

**Introduction:**

Understanding the association between risk factors and clinical outcomes of COVID-19 can lead to identifying suitable management strategies for reducing the mortality rate among maternal COVID-19 patients in the ICU.

**Aim of the Study:**

This study aims to investigate the clinical outcomes and risk factors associated with pregnant and postpartum women diagnosed with COVID-19 and admitted to the intensive care unit (ICU) between May 2020 and September 2021.

**Materials and Methods:**

This retrospective cohort study was conducted at the Universitas Indonesia Hospital. Secondary data was collected from the medical records to include all pregnant and postpartum women diagnosed with confirmed COVID-19 admitted to the hospital during the research period.

**Results:**

The study included 113 patients and found that admission to the ICU, age, and gestational age significantly influenced clinical outcomes, with a mortality rate of 42.11% among ICU-admitted patients. Pre-existing comorbidities such as type-2 diabetes mellitus, congestive heart failure, and coronary artery disease were associated with ICU admission. Having at least one comorbidity was found to increase the mortality rate by six-fold.

**Conclusions:**

The study emphasizes the importance of monitoring and evaluating maternal and fetal complications during COVID-19 infection, highlighting the need for multidisciplinary management involving intensivists, obstetricians, anesthesiologists, and infectious disease specialists. The findings underscore the significance of baseline health status in treatment planning and the potential for evidence-based interventions to improve maternal outcomes and pregnancy preservation. Further research is warranted to validate these results and enhance understanding of the underlying pathophysiology.

## Introduction

Coronavirus disease 2019 (COVID-19) is a viral respiratory illness with symptoms ranging from mild to critical. The first case of COVID-19 in Indonesia was confirmed on March 2^nd^, 2020, and though global numbers are dwindling, COVID-19 prevalence once reached 2.8 million per week in Indonesia in May 2021 [1]. The pandemic affected regular healthcare services nationwide, including maternal health services. One study in a referral hospital in Indonesia stated a 43.9% positivity rate among pregnant women suspected of COVID-19 infections [2].

Several studies have shown that viral respiratory illness during pregnancy negatively affects maternal and fetal outcomes [3,4]. Physiological changes in pregnancy may provoke changes in response to the infection, causing different manifestations of the disease, but the association is still unclear [5]. Indonesian Society of Obstetrics and Gynecology stated a 3% mortality of all pregnant women with COVID-19 from April 2020 – April 2021 in the country, just before a second wave of infection affected the country in May–August 2021 [6]. The maternal complication rate tended to be higher in women affected by COVID-19. Concerns about the impacts of COVID-19 on pregnant women arise based on the increasing morbidity and mortality rate compared to pre-pandemic conditions [2].

Mortality among pregnancy most often occurs in severe to critical patient groups with pre-existing comorbidities that make those pregnant women more vulnerable. Adverse outcomes were related to the higher risk of contracting more severe infections and the anxiety of accessing healthcare, which delayed complication identifications [7]. As an emerging infectious disease, understanding the association between risk factors and clinical outcomes leads to identifying suitable management for maternal COVID-19 patients in the ICU to reduce the mortality rate among those patients.

## Materials and Methods

This retrospective cohort study was conducted at the Universitas Indonesia Hospital, a university-affiliated hospital. Secondary data was collected from medical records to include patients hospitalized in the ICU from May 2020 to September 2021 after approval by the Ethics Committee of Universitas Indonesia Hospital (S-068/KETLIT/RSUI/XI/2021).

This study included all pregnant and postpartum women diagnosed with confirmed COVID-19 admitted during the research period. Data from 113 patients were collected from the registry and electronic medical records. Confirmed diagnosis of COVID-19 was defined according to the WHO case definition from the Public Health Surveillance document from December 2020 as patients with a probable or confirmed case who had tested positive in the nucleic acid amplification test or reverse transcription-polymerase chain reaction assays of COVID-19 infection and has symptoms or signs of anosmia or ageusia with no other known cause, severe acute respiratory illness, thorax imaging findings indicative of COVID-19, or death preceded by respiratory distress.

The severity of COVID-19 is grouped into mild, moderate, severe, and critical following the Indonesian COVID-19 Management Guidelines, as shown in Table 1 [8]. Patients that are admitted to the ICU are the ones with critical COVID-19 or other patients that according to the physician in charge's judgement are at high risk of developing critical conditions. Moreover, other variables indicating the severity of the infection include breathing mechanisms during stay in the ICU, use of invasive arterial blood pressure (ABP) monitoring, echocardiography and chest X-ray results, and several laboratory findings. Breathing mechanisms refer to oxygen ventilation methods: simple mask, non-rebreathing mask (NRM), high flow nasal cannula (HFNC), non-invasive ventilation (NIV), and ventilator, weaning off ventilation, prone position, and complication of pneumothorax. Echocardiography results involve normal or abnormal dimension, ejection fraction (EF) below or above 55%, tricuspid annular plane systolic excursion (TAPSE) indicators, presence of diastolic dysfunction, valve abnormality, and pericardial effusion. The radiographic results of the chest X-ray evaluate whether there are signs of below or above 50% bilateral infiltrate. Laboratory findings include Hemoglobin, Leukocytes, Thrombocytes, D-Dimer, procalcitonin, CRP, SGOT, SGPT, albumin, urea, creatinine, pH, PO2, pCO2, and PF ratio. Treatment of maternal COVID-19 patients in this ICU includes kidney replacement therapy (or no kidney replacement therapy), antiviral, corticosteroid, anticoagulant, IL-6 receptor antagonist, intravenous immunoglobulin (IVIG), and plasma convalescence therapy. Survival outcomes were observed during the ICU stay and grouped into clinical improvement or death.

**Table 1.** Criteria of adult COVID-19 severity classification according to the Indonesian COVID-19 Management Guidelines

**Severity Level**	**Criteria**
Mild	No evidence of pneumonia or hypoxia.
	Symptoms are limited to fever, cough, fatigue, anorexia, shortness of breath, myalgia, and/or other non-specific
	symptoms such as sore throat, nasal congestion, headache, diarrhea, nausea and vomiting, anosmia, or ageusia.
	SpO2 > 95% on room air.

Moderate	Clinical signs of pneumonia (fever, cough, dyspnea, tachypnea) with no sign of severe pneumonia.
	SpO2 ≥ 93% on room air.

Severe	Clinical signs of pneumonia (fever, cough, dyspnea, tachypnea) with at least one of the following: Respiratory rate > 30 breaths per minute, severe respiratory distress, and/or SpO2 < 93% on room air.

Critical	Patients with acute respiratory distress syndrome, sepsis and septic shock, or other conditions requiring external life support such as mechanical ventilation or vasopressor therapy.

SpO2, Peripheral oxygen saturation

Collected data was sorted in a computer database and analyzed using Statistical Package for the Social Sciences (SPSS) version 26.0. Characteristics of research subjects were analyzed descriptively. Normally distributed data are reported as means and standard deviations, while those not normally distributed are shown as medians with minimum and maximum. Normality was evaluated using the Saphiro-Wilk test. Two-sided statistical significance was set at p less than 0.05. Numerical data were analyzed using an independent T-Test or Mann-Whitney Test, while categorical data were analyzed using Pearson's chi-squared or Fisher's exact tests.

## Results

The total number of pregnant and postpartum women who were diagnosed and admitted for COVID-19 during this study period was 113 patients, and 19 (16.81%) patients required further care in the intensive care unit. The general characteristics of all 113 patients show that admission to the ICU, age, and gestational age were statistically significant in their associations with the clinical outcomes (p<0.001, p= 0.015, and p=0.029, respectively). All 94 patients who were only admitted to the general ward showed clinical improvement and recovery. Meanwhile, the mortality of those admitted to the ICU was 42.11%. Several pre-existing comorbidities were associated with the admission of our patients to the ICU, including type-2 diabetes mellitus (p=0.015), congestive heart failure (p=0.309), and coronary artery disease (p=0.027). No patients had chronic obstructive pulmonary disease (COPD), but two patients had asthma, which was found to be a statistically insignificant factor in ICU admission. Other preexisting conditions were found, such as post-chemotherapy nasopharyngeal cancer in one patient who did not survive and tuberculous lymphadenitis in one patient who survived.

Table 2 shows the demographic characteristics and comorbidities of 19 pregnant and postpartum patients with confirmed COVID-19 diagnosis admitted to the ICU. Two mild COVID-19 cases were admitted into the ICU after their Caesarean sections due to the presence of maternal complication preeclampsia and preexisting comorbidity of congestive heart failure that needed more intensive monitoring; however, both patients were able to recover. Despite COVID-19 severity being a substantial factor in the admission to the ICU, it was not found to be associated with eventual outcomes in the unit. No significant differences were observed among all of the other baseline characteristics or the presence of comorbidity in both groups.

**Table 2.** Baseline characteristics of maternal COVID-19 patients in ICU

**Characteristics**		**Outcomes**		**P value**
		**Improvement (N=11)**	**Death (N=8)**	
Age (years)		34±5	34±3	0.911
Gestational age (weeks)		30 (22–41)	32 (5–38)	0.901

Pregnancy status at admission				1.000
Pregnant		8 (61.5%)	5 (38.5%)	
Post delivery		3 (50%)	3 (50%)	

COVID-19 severity				**0.048**
Mild		2 (100%)	0 (0%)	
Severe		7 (77.8%)	2 (22.2%)	
Critical		2 (25%)	6 (75%)	
Length of stay (days)		9 (1–31)	10 (2–13)	0.901

Comorbidities				
Nasopharyngeal cancer				0.421
	Yes	0 (0%)	1 (100%)	
	No	11 (61.1%)	7 (38.9%)	
Congestive heart failure				0.546
	Yes	1 (33.3%)	2 (66.7%)	
	No	10 (62.5%)	6 (37.5%)	
Coronary artery disease				0.421
	Yes	0 (0%)	1 (100%)	
	No	11 (61.1%)	7 (38.9%)	
Chronic hypertension				0.421
	Yes	0 (0%)	1 (100%)	
	No	11 (61.1%)	7 (38.9%)	
Obesity				0.164
	Yes	0 (0%)	2 (100%)	
	No	11 (64.7%)	6 (35.3%)	
Asthma				1.000
	Yes	1 (100%)	0 (0%)	
	No	10 (55.6%)	8 (44.4%)	
Type-2 diabetes mellitus				1.000
	Yes	2 (66.7%)	1 (33.3%)	
	No	9 (56.2%)	7 (43.4%)	
Tuberculous lymphadenitis				1.000
	Yes	1 (100%)	0 (0%)	
	No	10 (55.6%)	8 (44.4%)	

Data are n (%), mean ± standard deviation, and median (minimum-maximum). COVID-19, coronavirus disease 2019

Table 3 shows some notable complications identified in our test subjects during our care. Acute kidney injury (p=0.003), liver injury (p<0.001), and sepsis (p=0.001) were significantly associated with ICU admission.

**Table 3.** Associations of complications with admission to ICU

**Complications**		**Admission**		**P value**
		**General ward (N= 94)**	**ICU (N= 19)**	
Acute kidney injury				**0.003**
	Yes	1 (20%)	4 (80%)	
	No	93 (86.1%)	15 (13.9%)	
Liver injury				**<0.001**
	Yes	0 (0%)	5 (100%)	
	No	94 (87.0%)	14 (13.0%)	
Sepsis				**0.001**
	Yes	0 (0%)	4 (100%)	
	No	94 (86.2%)	15 (13.8%)	
Gestational hypertension				**0.033**
	Yes	2 (40%)	3 (60%)	
	No	92 (85.2%)	16 (14.8%)	
Preeclampsia				0.526
	Yes	3 (75%)	1 (25%)	
	No	91 (83.5%)	18 (16.5%)	
Eclampsia				1.000
	Yes	1 (100%)	0 (0%)	
	No	93 (83.0%)	19 (17.0%)	

ICU, intensive care unit

Eleven patients gave birth at viable gestational ages (57.9%), one had a miscarriage at under 20 weeks gestation (5.3%), and seven continued the pregnancy until either clinical outcome was reached (36.8%). Patients needing intensive care were in respiratory distress, and their primary obstetric doctors usually recommended pregnancy termination. Intrauterine fetal death was observed in four pregnancies (36.4% of all terminated pregnancies), in which case one patient did a vaginal delivery as she was in labor at the point of ICU admission. All other patients underwent cesarean sections, with four patients receiving spinal anesthesia and six patients receiving general anesthesia with invasive ventilation. Another 36.4% of pregnancies ended preterm, and 27.2% of pregnancies carried their babies to term. None of these variables were significantly associated with final maternal outcomes.

Table 4 shows the risk factor of having at least one comorbidity of type-2 diabetes mellitus, hypertension, preeclampsia/eclampsia, congestive heart failure, peripartum cardiomyopathy, acute coronary syndrome, kidney injury, liver injury, asthma, or chronic obstructive pulmonary disease towards mortality outcome of all subjects. This study found that having at least one of said comorbidities was statistically significant to a six-fold mortality rate (p=0.026; OR=6.00; 95%CI=1.35–26.62).

**Table 4.** The Association between Comorbidities and Outcomes

**Comorbidity**	**Outcomes**		**P value**
	**Improvement (N=11)**	**Death (N=8)**	
At least 1	15 (78.9%)	4 (21.1%)	**0.026**
None	90 (95.7%)	4 (4.3%)	

Table 5 shows the ventilation support that our subjects received in the ICU. At baseline, the type of oxygenation therapy and the oxygen saturation levels at admission were statistically significant on patients' outcomes (p=0.014 and p=0.027). Moreover, all 19 subjects showed bilateral diffuse infiltrates on chest radiology. However, there was a statistically significant difference between the outcomes of patients with more or less than 50% infiltrates (p=0.013). All eight subjects who did not survive ICU stay were on invasive ventilators. The use of invasive ventilators (p<0.001), intubation time (p<0.001), and modes of ventilation (p=0.011) were found to be statistically significant factors in their eventual outcome. Subjects in prone positions showed less likelihood of a better clinical outcome, but the difference is statistically insignificant (OR=0.952 (0.144–6.281), p=1.000).

**Table 5.** Oxygenation support in the ICU

**Variables**	**Outcome**		**OR (95% CI)**	**P value**
	**Improvement (N = 11)**	**Death (N = 8)**		
**Baseline Respiratory Status**				
Oxygenation therapy at ICU admission				**0.014**

Simple mask	2 (100%)	0 (0%)	N/A	
Non-rebreathing mask	0 (0%)	1 (100%)	N/A	
HFNC	7 (70%)	3 (30%)	N/A	
NIV	2 (100%)	0 (0%)	N/A	
Ventilator	0 (0%)	4 (100%)	N/A	

Oxygen saturation levels at admission to the hospital	95.45±3.05	82.63±12.99	N/A	**0.027**

Chest X-ray				**0.013**
Bilateral infiltrate >50%	4 (33.3%)	8 (66.7%)	N/A	
Bilateral infiltrate <50%	7 (100%)	0 (0%)	N/A	

Mechanical Ventilation				

NIV (N = 8)	3 (37.5%)	5 (62.5%)	0.516 (0.916–1.353)	0.181
Invasive ventilators (N= 9)	1 (11.1%)	8 (88.9%)	N/A	**<0.001**

Intubation timing				**<0.001**
ER admission	0 (0%)	4 (100%)	N/A	
Day 1–2 in ICU	1 (50%)	1 (50%)	N/A	
≥ Day 3 in ICU	0 (0%)	3 (100%)	N/A	

Days on ventilator support	7	6.63+3.02	N/A	N/A

Pneumothorax on ventilator	0 (0%)	1(100%)	N/A	0.421

Prone positioning				1.000
Yes	4 (57.1%)	3 (42.9%)	0.952 (0.144–6.281)	
No	7 (58.3%)	5 (41.7%)	1.000	

Data are n (%), mean ± standard deviation, and median (minimum-maximum). ICU, intensive care unit; HFNC, high-flow nasal cannula; NIV, non-invasive ventilation; ER, emergency room

Table 6 shows the hemodynamic status of our subjects. During treatment in the ICU, most patients received vasopressors, mostly norepinephrine or dobutamine, and results show that it had significant associations with clinical outcomes (p=0.003). Meanwhile, the antiarrhythmic amiodarone was given to almost half of the subjects and was found to have statistically significant associations with clinical outcomes (p=0.005). Thirteen patients underwent echocardiography, but several patients did not report any examinations on TAPSE and valve abnormality, causing incomplete data. We found no difference in heart dimension and ejection fraction between the improving and the dead patients.

**Table 6.** Hemodynamic status in the ICU

**Variables**		**Outcomes**		**OR (95% CI)**	**P value**
		**Improvement (N = 11)**	**Death (N = 8)**		
**Hemodynamic status**					
Arterial line inserted					0.633
	Yes	6 (50%)	6 (50%)	0.400 (0.055–2.933)	
	No	5 (71.4%)	2 (28.6%)	1.000	

**Echocardiography (N = 13)**					
Dimension					1.000
	Abnormal	4 (44.4%)	5 (55.6%)	0.800 (0.076–8.474)	
	Normal	2 (50%)	2 (50%)	1.000	
Ejection fraction					1.000
	<55%	0 (0%)	1 (100%)	N/A	
	≥55%	6 (50%)	6 (50%)	N/A	
TAPSE (N=11)					N/A
	≤16	0	0		
	>16	5 (45.5%)	6 (54.5%)		
Diastolic dysfunction					1.000
	Yes	1 (33.3%)	2 (66.7%)	0.500 (0.034–7.452)	
	No	5 (50%)	5 (50%)	1.000	
Valve abnormality (N=12)					1.000
	Yes	4 (44.4%)	5 (55.6%)	0.800 (0.076–8.474)	
	No	1 (33.3%)	2 (66.7%)	1.000	
Pericardial effusion					1.000
	Yes	0 (0%)	1 (100%)	N/A	
	No	6 (50%)	6 (50%)	N/A	
Any vasopressor					**0.003**
	Yes	3 (27.3%)	8 (72.7%)	N/A	
	No	8 (100%)	0 (0%)	N/A	
Any antiarrhythmic					**0.005**
Yes		2 (22.2%)	7 (77.8%)	0.032 (0.002–0.426)	
No		9 (90%)	1 (10%)	1.000	

TAPSE, tricuspid annular plane systolic excursion

Table 7 shows the associations regarding leukocyte levels, C-reactive protein/CRP, SGOT, serum creatinine, blood pH, pCO2, and PF ratio, and clinical outcomes were statistically significant. However, several incomplete examination data or laboratory findings were reported for the research subjects. Blood tests were only routinely performed if there were indications for them.

**Table 7.** Laboratory findings on patients' clinical outcomes

**Laboratory parameters**	**Reference Values**	**Pregnancy Effect**	**Outcomes**		**P Value**
			**Improvement (N = 11)**	**Death (N = 8)**	
Haemoglobin	12.0 – 15.0 g/dL	1.4 – 2.0 g/dL decrease	10.10±1.54	9.63±1.84	0.549
Leukocytes	4.0 – 10.0 x 10^3^/μL	3.5 x 10^3^/μL increase	13.51 (5.20–46.34)	33.03 (6.17–67.30)	**0.032**
Thrombocytes	150 – 410 x 10^3^/μL	Slight decrease	248.74±149.46	210.00±149.57	0.584
D-Dimer	< 500.0 ng/mL	130 – 170 ng/mL	3107.46±2021.33	4248.74±2705.49	0.320
C-reactive protein	<5.0 mg/L	0.4 – 8.1 mg/L	83.71±67.60	148.03±40.38	**0.031**
Procalcitonin	<0.05 ng/mL	-	0.20 (0.20–0.74)	0.70 (0.20–7.13)	0.074
SGOT	0 – 31 U/L	-	36.00 (17.00–230.00)	63.00 (37.00–309.00)	**0.028**
SGPT	0 – 31 U/L	-	36.50 (12.00–230.00)	31.00 (22.00–379.00)	0.770
Albumin	3.2 – 4.6 g/dL	1 g/dL decrease	3.31±0.53	3.08±0.33	0.434
Urea	15 –40 mg/dL	50% decrease	22.00 (12.00–141.00)	66.00 (14.00–243.00)	0.126
Serum creatinine	0.50 –1.00 mg/dL	-	0.57 (0.41–1.38)	1.12 (0.56–7.99)	**0.026**
Arterial blood gas					
pH	7.350 – 7.450	-	7.36±0.10	7.13±0.17	**0.019**
pO_2_	75.00 – 100.00	-	80.70 (56.00–268.00)	68.25 (57.50–118.40)	0.307
pCO_2_	35.00 – 45.00	25.00 – 33.00	38.70±9.06	59.67±13.53	**0.007**
P/F Ratio	>300	-	127.09 (70–382.86)	68.25 (57.50–118.40)	**0.006**

Data are n (%), mean ± standard deviation, and median (minimum-maximum). Pregnancy effects listed were for third-trimester changes if there were differences between the trimesters. Reference: Abbassi-Ghanavati M, Greer LG, Cunningham FG. Pregnancy and laboratory studies: a reference table for clinicians https://www.openanesthesia.org/keywords/pregnancy_laboratory_measurements/

Table 8 shows receiving kidney replacement therapy was associated with mortality, and patients without renal support mostly showed clinical improvement (p=0.031). Numbers of analgesics and sedatives administered were significantly related to the outcomes of research subjects (p<0.001). Subjects who received anticoagulants show numerically higher rates of clinical improvement and survival despite no statistical significance (OR = 3.333 (0.246–45.109)), p = 0.546)).

**Table 8.** Treatments in the ICU

**Treatment**	**Outcomes**		**OR (95% CI)**	**P**
	**Improvement (N = 11)**	**Death (N = 8)**		
Kidney replacement				**0.031**
Hemodialysis	0 (0%)	2 (100%)	N/A	
CRRT	0 (0%)	2 (100%)	N/A	
No	11 (73.3%)	4 (26.7%)	N/A	

Any analgetic-sedation				**<0.001**
None given	8 (100%)	0 (0%)	N/A	
1–3 medications	3 (60%)	2 (40%)	N/A	
>3 medications	0 (0%)	6 (100%)	N/A	

Antiviral				0.444
Remdesivir	10 (58.8%)	7 (41.2%)	N/A	
Remdesivir and Oseltamivir	0 (0%)	1 (100%)	N/A	

Corticosteroid				0.251
Dexamethasone	7 (63.6%)	4 (36.4%)	N/A	
Methylprednisolone	0 (0%)	3 (100%)	N/A	
Both	2 (66.7%)	1 (33.3%)	N/A	

Anticoagulant				0.546
Yes	10 (62.5%)	6 (37.5%)	3.333 (0.246–45.109)	
No	1 (33.3%)	2 (66.7%)	1.000	

Tocilizumab (Actemra)				0.319
Yes	2 (33.3%)	4 (66.7%)	0.222 (0.028–1.754)	
No	9 (69.2%)	4 (30.8%)	1.000	

Intravenous immunoglobulin				0.421
Yes	0 (0%)	1 (100%)	N/A	
No	11 (61.1%)	7 (28.9%)	N/A	

Convalescent plasma				0.319
Yes	2 (33.3%)	4 (66.7%)	0.222 (0.028–1.754)	
No	9 (69.2%)	4 (30.8%)	1.000	

CRRT, continuous renal replacement therapy

## Discussion

In this report, 16.81% of 113 pregnant and postpartum patients who tested positive for COVID-19 were admitted to the ICU. Other reports early in the pandemic showed a considerably lower rate of maternal ICU admission at 3–10% [9,10,11]. However, previous studies have found associations between a higher risk of infection and more severe forms of the disease at later gestational ages [9,10,12]. Most of our patients were women in the third trimester of their pregnancies, which may contribute to the higher ICU admission rate. Our analysis also identified a significant association between the development of several systemic complications throughout disease progression and the likelihood of ICU admissions, with these complications being linked to deteriorating clinical conditions.

Our study found that the mortality rate of pregnant COVID-19 patients admitted to the ICU was very similar to the findings of the study conducted in Turkey by Eman et al. [12]. We found that among all admitted patients, the mortality rate was 7.08%. However, another study in Germany found a much lower ICU mortality rate of 5%. This finding may be attributed to the different criteria of ICU admission, as the said research also admitted patients with mild and moderate COVID-19 symptoms to the ICU for observation. [9]

There was a significant association with maternal mortality in patients admitted to the ICU, which was self-explanatory considering the differing degrees of severity between the two groups, while severe and critical patients were mainly admitted to the critical unit. Previous studies suggested that COVID-19 appeared less lethal than SARS and MERS infections, and most infected patients had little to mild symptoms [4]. However, as in the general population, maternal patients with underlying conditions had higher chances of more severe disease progression [4]. In our study, several systemic comorbidities had significant associations with admission to the critical unit but not with survival outcomes in the ICU. This suggests that the degree of COVID-19 infections significantly influenced mortality more than present pre-existing diseases for our study subjects.

All 19 patients needed some form of oxygenation support at the point of ICU admission, as oxygen saturation levels were highest at only 95%. To accommodate the semi-allogeneic fetus, the pregnant body decreases the reactivity of cell-mediated immunity responses, diminishing the body's ability to clear viral infections and making it more vulnerable to severe infections [13]. This further solidifies the theory that pregnancy, on its own, can be considered a risk factor for poor COVID-19 prognosis. This cohort did not stratify disease severity according to specific gestational ages, and further research is needed to identify riskier populations who may benefit from earlier or more aggressive therapies.

In our research, we did not find any specific comorbidity that increases the mortality rate or risk for ICU admission in pregnant COVID-19 patients. Nevertheless, having at least a comorbidity increases the risk of death in ICU patients. This finding is common sense and is consistent with all previous studies. [9,10,12] A study by Mustafa et al. stated that comorbidities such as obesity, diabetes, cardiovascular disease, hypertension, and immune impairment are direct risk factors for COVID-19 maternal mortality. [14]

During ICU stay, nine patients (47.37%) required invasive ventilation, which was significantly associated with worse survival rates. This finding aligns with previous reports that showed pregnant women to be more significantly likely to require ventilation support and endotracheal intubation compared to their counterparts [5]. We also applied intermittent prone positioning to 36.84% of patients across the different ventilation support groups, with results suggesting no statistical association with the clinical outcomes. Other studies found that prone positioning was beneficial and indicated in patients with severe hypoxemia [3,15]. However, similar to the findings of a study by Eman et al., our results suggested that this position did not negatively affect the need for more invasive ventilation means.

Despite that, poor echocardiographic findings were always found at numerically higher rates in the non-surviving group, suggesting that complications to the heart function could be a factor for our mortality. In their report, Afari HA et al. reviewed several normative changes to echocardiography in pregnancy, which included reversible dilation of heart chambers, tricuspid and pulmonic valve regurgitation, and asymptomatic pericardial effusion. However, lower EF and diastolic dysfunction were never normal findings.

Moreover, echocardiography was essential in evaluating the right ventricle functions in suspicion of acute cor-pulmonale [16]. Our study found that TAPSE scores were normal for all evaluated patients; thus, in these cases, cardiomyopathies were more likely independent heart complications to the infection and not related to cor-pulmonale.

While we did not investigate simultaneous bacterial infection in this study, our findings showed that sepsis was only found in subjects admitted to the ICU. This study also observed statistically significant increased total white blood cells and CRP in non-surviving patients. Previous studies have described CRP as an important inflammatory biomarker in COVID-19 disease progression, as systemic inflammation is behind the severity of infection. Our results were in agreement with previous reports that showed leukocytosis and high levels of CRP in association with disease morbidity and mortality [11,17].

D-dimer values were considerably high in both groups, but no associations with clinical outcomes were found, although previous evidence reported it as a poor prognostic factor [11,18]. Nevertheless, a physiologic increase of D-dimer is remarkably seen even in healthy pregnant women.

By identifying complications in this study, significant laboratory findings on kidney and liver functions, namely SGOT and serum creatinine, reflected worse organ injury in the non-surviving group. To begin infection, the SARS-CoV-2 virus attaches itself to angiotensin-converting enzyme-2 receptors, which are coincidentally expressed in numerous body parts, including renal tubules and hepatocytes [19].

Acute kidney injury (AKI) is found in a quarter of the critically ill nonpregnant population, and the risk increases by 51% during pregnancy [20]. Our finding of only one preeclampsia case in the ICU contradicts the literature, which reported that the incidence of pregnancy-related AKI was usually found in conjunction with preeclampsia with severe features or HELLP syndrome [20]. This is thought to be related to the endothelial disruption-related pathophysiologies in both disease entities. Despite that, tubular renal damage may also be directly caused by the virus or through the effects of cytokine storm [19], which may be more applicable in our cases. This suggests that renal function evaluation should not be exclusive to patients with signs, history, or risks of preeclampsia and that blood pressure monitoring is an easy and invaluable tool in first screening for AKI case finding.

Sekulovski et al. reported that COVID-19-related liver dysfunction was more commonly identified in patients with severe or critical disease, matching our findings that all five cases of liver failure were in subjects admitted to the ICU. As in kidney injuries, liver damage is postulated to be related to an impaired immune response to the viral infection or due to severe hypoxia. Studies found that these patients would have higher levels of procalcitonin and liver enzymes compared to infected pregnant women without liver injury [20,21]. Our study found that the elevation of liver enzyme (SGOT) and the number of analgesic medications administered were significantly higher in the non-surviving group. However, the correlation between the two still needs to be investigated. [20,21]. This enforces the importance of liver function monitoring in infected pregnant patients treated with multiple medications.

In the non-surviving group, blood gas results showed significantly lower blood pH, more severe hypercapnia, and lower PF ratio. Non-pregnant patients with ARDS are often put in hypercapnia conditions to decrease the risk of injury due to (high) tidal volumes. However, during pregnancy, the reference value of PaCO_2_ level is lower due to adapted physiology, and high PaCO_2_ may be associated with respiratory acidosis in fetuses. This causes a right shift on the fetal hemoglobin oxygen dissociation curve, thus negating the higher affinity to oxygen characteristic of fetal hemoglobin [3]. Additionally, our patients demonstrated severe hypoxemia, which, although insignificant, allowed for theoretically even worse fetal outcomes. This study did not evaluate the effects of ARDS severity on newborns. Still, our findings were consistent with other reports, which showed the associations of severe respiratory distress with preterm births, in which our critical patients had more premature termination of pregnancies rather than term laborers [18].

Our study revealed no significant differences in administered medicine, aside from symptomatic analgesic/sedation medications, between clinical outcome groups. This finding is similar to a prior study conducted in the United States, stating that the use of higher benzodiazepine doses was associated with increased mortality in mechanically ventilated patients [22]. This may be caused by the need for more analgesia and sedation to facilitate comfort and achieve ventilator tolerance in patients with more severe respiratory distress.

The main limitations of this study were the small sample size and single-center design. This was also a retrospective cohort study that depended only on available information on the medical records, which may cause bias due to the incompleteness of data. This problem arises from the lack of a study protocol that may dictate standard data collection as well as laboratory and radiological examinations at a specified time. Nevertheless, this research allowed for the analysis of extensive data on the ICU treatment of COVID-19 in a specific population. Further studies on this or similar topics with a controlled prospective study protocol would be beneficial in providing more accurate data.

## Conclusion

Admission to ICU for severe and critical COVID-19 in pregnant and postpartum patients was associated with mortality and poor prognosis. The presence of preexisting comorbidities was associated with requiring more intervention but was not related to outcome. Several maternal and fetal complications were identified during the disease, and constant monitoring and evaluation are principal to optimize necessary care. Baseline health status should be an essential part of the treatment plan, especially respiratory system-related ones. Management of these patients should be multi-disciplinary and include approaches from intensivists, obstetricians, anesthesiologists, and infectious disease specialists, as adequate and evidence-based treatments may lead to complete maternal recovery and conservation of pregnancy.
